# The Strategies for Quantitative and Qualitative Remote Data Collection: Lessons From the COVID-19 Pandemic

**DOI:** 10.2196/30055

**Published:** 2022-04-08

**Authors:** Keenae Tiersma, Mira Reichman, Paula J Popok, Zoe Nelson, Maura Barry, A Rani Elwy, Efrén J Flores, Kelly E Irwin, Ana-Maria Vranceanu

**Affiliations:** 1 Department of Radiology Massachusetts General Hospital Boston, MA United States; 2 Department of Psychiatric Oncology Massachusetts General Hospital Boston, MA United States; 3 Integrated Brain Health Clinical and Research Program Department of Psychiatry Massachusetts General Hospital Boston, MA United States; 4 Implementation Science Core Department of Psychiatry and Human Behavior Brown University Providence, RI United States; 5 Center for Healthcare Organization and Implementation Research VA Bedford Healthcare System Bedford, MA United States

**Keywords:** web-based research, remote research, remote data collection, blended design, electronic data collection, mobile phone

## Abstract

The COVID-19 pandemic has necessitated a rapid shift to web-based or blended design models for both ongoing and future clinical research activities. Research conducted virtually not only has the potential to increase the patient-centeredness of clinical research but may also further widen existing disparities in research participation among underrepresented individuals. In this viewpoint, we discuss practical strategies for quantitative and qualitative remote research data collection based on previous literature and our own ongoing clinical research to overcome challenges presented by the shift to remote data collection. We aim to contribute to and catalyze the dissemination of best practices related to remote data collection methodologies to address the opportunities presented by this shift and develop strategies for inclusive research.

## Introduction

### Background

The COVID-19 pandemic is transforming the landscape of clinical research. The pandemic has necessitated the unexpected adaptation of ongoing clinical research activities to web-based or blended design (ie, part web-based, part in-person) models [1] and has rapidly accelerated a shift within clinical research toward web-based study designs. Despite the high levels of patient and health care provider satisfaction with telemedicine and virtually conducted clinical research [2,3], many challenges exist to the web-based conduct of rigorous, efficient, and patient-centered clinical research, particularly related to the engagement of diverse and marginalized populations [4]. The aim of this paper is to discuss practical strategies to guide researchers in the remote collection of quantitative and qualitative data, derived from both previous literature and our own ongoing clinical research.

Many health care providers and clinical researchers have marveled at the way the COVID-19 pandemic catalyzed the widespread adoption and expansion of telemedicine, seemingly overnight [5,6]. Despite the sluggish adoption of telemedicine observed in academic medical centers over the past several decades [7,8], the pandemic has spurred rapid changes in public and organizational policy regulating telemedicine in the United States, facilitating a tipping point toward the web-based provision of both health care and conduct of clinical research [1,2,5,6]. Enabled by fast-tracked institutional review board policies and amendments [1], researchers have adapted clinical research study procedures in innovative ways: engaging in web-based outreach for study recruitment, collecting electronic informed consent, conducting study visits, delivering interventions over the phone or live video, and using remote methods to collect data [1]. Several studies have reported high satisfaction of both providers and patients with the use of telemedicine during COVID-19 and a willingness to continue using telemedicine after the pandemic, including for clinical research [2,3].

This shift toward virtually conducted clinical research creates many *opportunities* to increase the accessibility of clinical research. Virtually conducted research reduces many burdens on patients associated with research participation, including time and monetary costs involved in travel to research facilities. This enables researchers to include patients who lack access to transportation or the ability to travel independently. Furthermore, web-based patient outreach allows researchers to recruit geographically diverse participants, enabling researchers to target populations through disease-specific registries, internet-based patient communities, and advocacy groups without geographical constraints [9]. By centering patients rather than investigative sites in the study design and operation, virtually conducted research has the potential to increase the patient-centeredness of clinical research [9].

At the same time, the transition to virtually conducted clinical research also presents many *challenges* to patient engagement and data collection. Losing supervision of the physical setting of research activities challenges researchers’ ability to ensure patients’ adherence to study protocols, engagement and interest in research activities, and privacy protections. Researchers are faced with complex decisions regarding the appropriateness of data collection methodologies or specific measures and assessments for web-based delivery [10]. Furthermore, there are barriers associated with the technology used for remote data collection (eg, telephones, electronic databases, live videoconferencing software, and ecological momentary assessment), including a lack of technology literacy and challenges using technology among both patients and research staff [1,4,8,11]. Finally, some patients lack access to smartphones, the internet, or secure and stable housing, which may preclude their participation in web-based clinical research unless researchers can allocate funding to provide these devices. Consequently, the transition to virtually conducted clinical research may further marginalize people in low-income and rural settings [4].

### Objective

To thoughtfully respond to the challenges associated with remote data collection and ensure that disparities in access to clinical research do not widen, there is a critical need for practical strategies for researchers. By integrating recommendations from previous literature with examples from the ongoing clinical research projects of this authorship team with extensive patient and provider populations (ie, adults and adolescents with neurofibromatosis, older adults with chronic pain and cognitive decline, adults with cancer and serious mental illness, adults with young-onset dementia, and orthopedic medical providers), we present a discussion of practical strategies for researchers to support the rigorous, efficient, and patient-centered collection of quantitative and qualitative data remotely. Summary tables present a list of strategies related to the remote collection of quantitative (Table 1) and qualitative (Table 2) data.

**Table 1:** Challenges in remote quantitative data collection and associated strategies.

Challenges	Strategy	Example approach
Study staff do not have sufficient technological experience or access to technology.	Equip study staff with technology as necessary and instruct them on foundational technological skills.	Ensure that all study staff have access to necessary technology to carry out study responsibilities (eg, laptops with webcams, phones, and software programs) and have been thoroughly trained in their use.
Validating participant credentials and ensuring data quality.	Incorporate eligibility, attention, and manipulation checks throughout surveys.	Attention check: for quality assurance, please select *strongly disagree* for this line. Manipulation check: who was in the video you just watched?
Study staff lack experience remotely communicating with study participants.	Train study staff on good clinical practices and foundations of verbal and nonverbal communication that are appropriate for web-based setting.	Study staff collecting measures should be educated on best practices for protecting participant privacy and confidentiality through remote methods (ie, use of secure software and calling from private locations). Study staff should be trained on verbal and nonverbal communication appropriate for web-based settings (eg, eye contact through webcams and body language from shoulders up) and be mentored with peer or hierarchical supervision.
Managing the secure electronic distribution of measures to study participants.	Use a secure web platform to distribute measures.	Study staff can use secure electronic platforms (eg, REDCap^a^ and Qualtrics^b^) to distribute measures and use functionalities (eg, scheduling surveys and automatic reminders) to maximize efficiency and organization for study team.
Assisting study participants in using technology to complete remote study measures.	Proactively identify participants’ comfort with technology and then tailor individualized supportive approaches.	Study staff should first engage in *meeting participants where they are* by determining participants’ technology comfortability and then supply participants with training accordingly (eg, written instructions, prerecorded videos, and live assistance). Study staff may also coordinate with members of the participants’ household to collaboratively support them with technology as needed.
Engaging participants who lack access to technology or lack technological literacy to independently complete remote study measures.	Allow flexible and multimodal alternatives for measurement completion, along with offering relevant instructions on using these modalities.	Study participants can be mailed letter copies of the self-report measures or give their answer to survey questions via phone calls with study staff. Study staff should communicate with study participants about their preferred modality and support with associated burden.
Adapting study protocol to determine new ways to gather non–self-report data that previously required in-person assessment.	Conduct a literature search to identify and implement innovative, creative, and flexible alternatives.	Study teams can use previously adapted and validated measures for remote delivery, such as a mobile app to measure distance walked in 6 minutes (ie, 6-minute walk test).
Burden on participants of completing remote electronic study measures.	Asynchronous distribution of study measures.	Study staff can send participants a link from a secure web platform so that participants can complete the measurement independently at a time and place most convenient for them.
Participants who require or prefer assistance in measure completion.	Provide live assistance to participants during measure completion (ie, synchronous completion) via phone or live video.	Pay attention to participants’ focus, engagement, and comprehension during synchronous measure completion (eg, ask participants if they have any questions about the phrasing of measures and offer participants the option to take pauses during the assessment).
Participants who require or prefer visual aid during synchronous measure completion.	Use *screen share* functions of HIPAA^c^-compliant videoconferencing technologies.	Using secure and institutionally approved videoconferencing technology (eg, Zoom and WebEx), study staff can *screen share* the survey so that patients can see, read, and potentially better comprehend questions.
Protecting participant privacy and confidentiality during remote calls.	Proactively promote actions in coordination with the study participant to uphold good clinical practice and protect privacy and confidentiality.	Confirm with study participants if they are in a safe space to openly answer questions, advising them of the sensitive nature of questions before administering measures, and assisting participants with strategies to maximize their privacy (eg, scheduling calls at a participant’s preferred time and wearing headphones).
Building rapport with study participants while communicating virtually.	Focus on body language, tone of voice, and language appropriate for web-based settings.	Use verbal strategies appropriate for web-based settings (eg, establish a conversational tone, use participants’ names, speak clearly and directly into microphone, and provide technological support so that participants feel comfortable) and nonverbal strategies appropriate for web-based settings (eg, smile at participants, make direct eye contact with webcam, and sit upright).
Promoting completion of unfinished surveys.	Schedule reminders at predetermined intervals for participants who have not completed measures.	Participants who do not complete the survey in a scheduled time can be prompted to do so via automated electronic reminders within the distribution platform or individual outreach (eg, calling, texting, reminding in person). Study staff should flexibly use different outreach methods and communicate in advance to the participant how often they will send reminders through each method.
Reaching participants who are difficult to reach via technological means or who are no longer responding to outreach.	Determine standardized study procedures about who will contact the participant, how many times, and through what method.	Decide on a number of times to call a participant before transferring the matter via an established chain of command. Use creative approaches such as considering the individual’s circumstances and best ways and times to reach them, involving family members, and involving incentives as appropriate.

^a^REDCap: Research Electronic Data Capture (Vanderbilt University).

^b^Qualtrics Survey Distribution (Qualtrics XM Platform).

^c^HIPAA: Health Insurance Portability and Accountability Act.

**Table 2:** Challenges in remote qualitative data collection and associated strategies.

Challenges	Strategies	Example approach
Inclusive outreach to participants.	Multimodal outreach strategies.	Send physical letters with focus group information and offer both telephone only and videoconferencing modalities.
Coordinating a meeting time for web-based interviews or focus groups.	Provide flexible hours and focus on participants’ schedule preferences.	Study staff can offer multiple times for web-based focus groups to assess times that would maximize attendance.
Securely conducting web-based interviews or focus groups.	Select HIPAA^a^-compliant videoconferencing platforms.	Platforms that are HIPAA-compliant have security features such as password-protected meetings and waiting rooms that study staff can use to protect participants’ privacy and confidentiality.
Encouraging active participation in web-based interviews or focus groups.	Proactively plan the focus group structure to optimize participation.	Consider the target size of the focus group to promote participation. Study staff can also give an introduction at the start of focus sessions to set a tone of welcome and inclusivity (eg, build rapport and give overview of study topics) and be intentional about the use of verbal and nonverbal communication throughout the focus group to encourage participation.
Solving technological issues with participants.	Review technological features and any problems at the start of the session and then address emerging issues as needed.	Expend a portion of the focus group, ensuring that all technological components are functioning (eg, microphones and videos turned on as appropriate) and review features of the platform. Have a study staff member on call to assist with technological problems as needed.
Conducting interviews or focus groups in a timely fashion.	Coordinate the team approach to adhering to a predetermined schedule.	Determine allotted time for aspects of the focus group’s discussion in advance and divide labor among the study staff during focus groups to maximize efficiency.

^a^HIPAA: Health Insurance Portability and Accountability Act.

## Strategies for Remote Data Collection

### Optimizing Quantitative Measures for Effective Remote Distribution and Delivery

Asynchronous distribution and measure completion (eg, electronic distribution of surveys) maximize efficiency for the study team and flexibility for study participants but necessitates additional consideration for participants with varying levels of familiarity with and access to technology. Secure web platforms (eg, REDCap [Research Electronic Data Capture], Vanderbilt University and Qualtrics, Qualtrics XM Platform) are ideal for asynchronous distribution because they have functionalities that promote study team efficiency and organization (eg, scheduling survey distribution in advance and automatic reminders to participants to complete surveys) while enabling participants to complete measures independently and at a time most convenient for them [9]. Although these platforms are widely compliant with Health Insurance Portability and Accountability Act (HIPAA) and regulatory requirements, study teams should ensure that platforms are compliant with institution-specific clinical research regulatory requirements before use (and consider potential differences between clinical research and clinical care requirements).

Many of these platforms also offer participant screening, consenting functionality, and mobile device compatibility, which maximize the utility for study teams [9]. Study teams relying on web-based platforms and asynchronous measure completion should also consider the adoption of flexible alternative options for measure completion to maximize completion rates and the engagement of participants. For example, study teams might offer participants the option to complete measures on paper through physically mailed surveys or over the phone with a member of the study staff, depending on participant technology access and preference. Similarly, in addition to electronic reminders integrated within the distribution platform, study teams will likely need to use other methods to contact participants and remind them to complete measures (eg, calling, texting, and reminding in person). To decrease the burden on participants and increase adherence to study procedures, participants should be informed of how many of these reminders to expect.

Validating participant credentials in studies where research staff have no personal interaction with participants (ie, web-based survey studies) is another challenge with web-based research. Data quality checks, such as eligibility, attention, and manipulation checks (see Table 1 for examples) can be introduced to protect from duplicate responses or participants falsifying information. Enabling IP address tracking is another feature of some survey platforms (eg, Qualtrics). As with all data collection, it is imperative that participants are aware of how their information is being collected and researchers have been granted previous institutional review board approval.

We use REDCap and rely on predominantly asynchronous measure completion to collect quantitative data in an ongoing randomized controlled trial of a mind-body intervention to promote quality of life in adults with a genetic condition called neurofibromatosis [12]. Participants receive links via email to complete surveys at all time points (ie, baseline, posttest, and 6- and 12-month follow-ups), and we set automatic email reminders to go out at defined intervals every 3 days until participants complete surveys. The frequency of reminders should be determined by the study team to balance the burden on study staff and participants with the desire to have high survey completion rates. We find that participants enjoy the flexibility of completing measures at their convenience from the comfort of their homes and using personal devices.

For quantitative measures other than self-report surveys, study teams may need to use innovative methods to adapt data collection methods for remote delivery. Although not all measures can be adapted for remote delivery (eg, imaging data collection), many can through a combination of creative and flexible strategies, including using mobile device data collection, mailing materials and devices to participants, and conducting assessments over live videoconferencing. Even the collection of biomarker data, common in quantitative research clinical trials, can sometimes be adapted for remote conduct through mailing of devices and use of smartphone technology, such as mailing saliva or nicotine strips for the verification of tobacco abstinence or the provision of personal devices to measure expired carbon monoxide that are compatible with smartphones [1]. In adapting measures for remote delivery, it is essential to examine previous literature to assess the availability of remote alternatives and evidence to support the validity of remote alternatives or adaptations [10]. Study teams’ attention to usability and patient burden is essential [10]. It may also be important to account for the modality of data collection during data analysis (eg, evaluating whether the mode of data collection is a confounder in multimodal studies).

In our randomized controlled trial with patients with chronic pain and cognitive decline, we conducted a literature search to identify remote methods for assessing cognitive functioning as well as performance-based physical function. The Montreal Cognitive Assessment [13], a measure we previously used in our in-person study [14], has been adapted and validated for remote administration over live videoconferencing [15,16]. Accordingly, we developed a standardized protocol for applying the Montreal Cognitive Assessment audiovisual procedures, including mailing participants a paper with items that required drawing and instructing them to display the paper to the camera for us to screenshot over videoconferencing [17]. Our literature review also identified a validated, free-of-charge mobile app that uses GPS coordinates to measure the distance walked in 6 minutes to replace the 6-minute walk test (6MWT) [18] that we had previously conducted in our in-person study [14]. Before using the app with participants, we piloted the app and developed a standardized protocol to assist participants in downloading the app, using the app, and reporting the results [17].

In the process of adapting quantitative measures for remote completion, the safety of the participants must be considered. For example, in our randomized controlled trial with adults with neurofibromatosis, we used the Patient Health Questionnaire-9, which contains an item assessing suicidal ideation, to measure depression. We developed a standardized protocol to respond to cases in which participants endorse suicidality, including collecting the name and number of an emergency contact for each study participant during enrollment, having the study clinician and principal investigator receive immediate notification from REDCap, and having the study clinician or principal investigator follow up over phone with the participant within 24 hours to complete a safety assessment [12]. Similarly, in our randomized controlled trial with older adults with chronic pain and cognitive decline, we considered the safety risks associated with asking participants to complete the 6MWT independently (eg, falls). Participants were asked to create a plan to complete the 6MWT on a familiar route at a designated date and time, with support from a friend or family member when possible [17].

### Synchronously Assisting Participants in Remote Completion of Quantitative Measures

Depending on the study protocol and population, the best practice may be the synchronous completion of measures (ie, in which a study team member administers the assessment to the participant in real time). The synchronous completion of self-report measures enables study staff to directly support participants in completing measures, including ensuring participants’ best effort, attention, focus, and comprehension during measure completion. Assisting participants synchronously in completing self-report measures also allows study staff to ensure that data are supplied directly from intended participants and eliminate the possibility that participants are being influenced by others such as spouses or parents. The factors to consider when making this decision include participants’ age, cognitive ability, previous experience with technology, and preference. When assisting participants with assessment completion remotely, multiple modalities that can be used. First, calling participants by phone requires minimal technology access and familiarity for participants and enables study staff to *catch* participants at an opportune moment and ensure prompt survey completion with minimal effort on the part of the participant. Over the phone, study staff can ensure comprehension of every item (important for data validity); however, reading aloud every question-and-answer option can also be tedious for both study staff and participants. Strategies to address comprehension and focus include pausing to ask if clarification is needed, breaking up longer questions, and asking participants if they wish to take a break throughout the conversation.

For some participants, the visual component was beneficial for enhancing their comprehension of measure items. Video calling a participant with HIPAA-compliant, secure platforms [1] (eg, with Zoom and WebEx) and *screen sharing* the measure is a novel strategy to support participants in completing measures remotely. This *screen share* method provides the opportunity for the participant to see the questions in addition to hearing them and can enable better comprehension as well as more efficient measure completion (eg, study staff may not need to read every answer choice for items when participants can read them on-screen). Mailing participants paper copies of surveys in advance of phone calls is another method for allowing participants to have questions in front of them while also receiving live assistance in responding.

We use this novel *screen share* strategy in an ongoing randomized controlled trial of a mind-body intervention to promote quality of life in geographically diverse adolescents aged 12 to 17 years with neurofibromatosis [19]. We decided to rely on synchronous measure completion for this population, given the age of participants and high rates of learning disabilities, leading to anticipated challenges with thoughtful independent measure completion, as well as anticipated challenges with comprehension of items. The method has been effective in engaging participants during data collection to ensure participant comprehension of items and thoughtfulness when selecting answer choices. This method has also allowed us to identify and eliminate situations in which participants’ parents are inappropriately coaching participants during data collection. Notably, videoconferencing does require a higher level of access and familiarity with technology; therefore, creative problem-solving abilities with participants are essential. As with all forms of technology used in data collection, study teams should consider ease of use for participants and be prepared to provide both emotional and technical support [11].

For group-based interventional studies and situations in which study staff want to be available to answer potential questions related to measure completion (about either technology use or specific items) but do not want to walk participants through every item, a group support procedure could be used using videoconferencing. In this strategy, a member of study staff can email participants the links to complete surveys on their own devices and schedule a time in which the group of participants joins a videoconferencing call to complete the measures at the same time. We use this strategy in our randomized controlled trial for older adults with chronic pain and cognitive decline [17]. Participants in a group video call are supported in navigating to their email to open the secure link to complete the questionnaires. Although completing their questionnaires independently, participants turn their video on or off, and we mute all participants and the study staff host to enhance focus and privacy and to replicate an in-person visit [14,17]. This method allows us to assist as needed when a participant takes themselves off mute to ask a question, physically raises their hand, or privately chats us. In addition, we periodically ask if anyone needs assistance, particularly after noticing that participants are not progressing as expected because REDCap allows the ability to monitor progress in real time.

As with the shift to remote clinical care, the privacy and confidentiality of patients is not as easy to ensure as it is in person. Research staff have an obligation to ensure participant privacy and confidentiality to adhere to the principles of good clinical practice [20] and to ensure the acceptability of study procedures to participants for whom concerns of being overheard are common [11]. Informing (or reminding) participants of the sensitive nature of the questions (eg, pertaining to physical health, mental health, and intimate relationships) and ensuring that they are in a space where they feel comfortable to answer is the best practice. Working with participants to ensure the highest level of privacy may be necessary. Suggestions include using headphones (both participants and research staff), inquiring about participants’ location and privacy, and allowing participants to determine the best time for the call [5,11]. Additional safety protocols are necessary when providing devices to participants, as they could be exposed to data theft or lose track of the device. We suggest enabling password protection on devices and limiting the data stored on the actual device to protect patient safety. Ultimately, although providing devices introduces the risk of needing to potentially replace the hardware, it is a readily integrable strategy to address the digital divide and increase access to research [21]. Participants should be reminded of the privacy risks associated with remote study participation (eg, possible breaches to the security of data collected remotely) and informed of the measures study staff are taking to safeguard against these risks (eg, encryption of devices and deidentification of data).

### Motivating Participants to Complete Quantitative Measures Remotely

**Figure 1:**
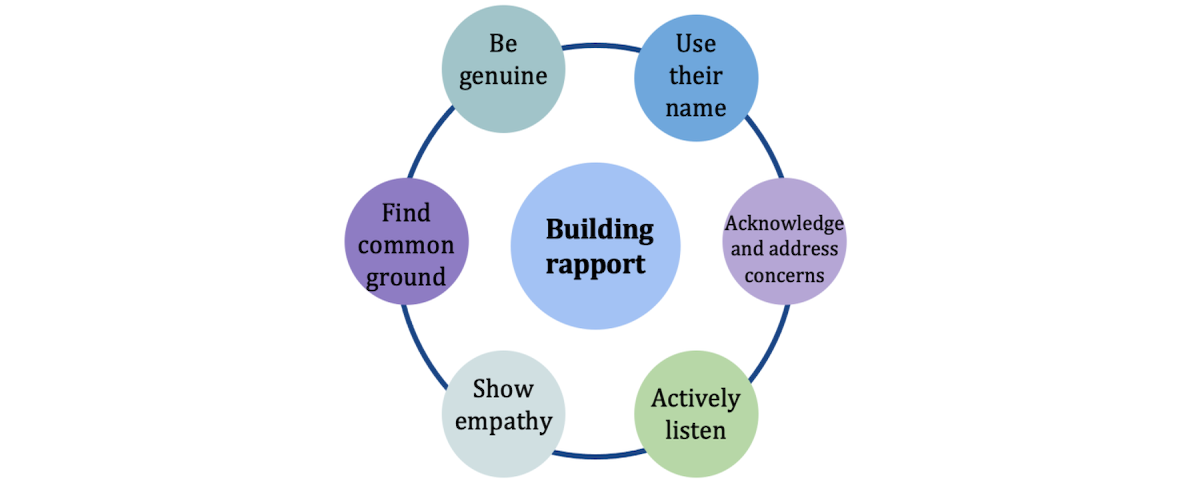
Building rapport.

Building relationships with study participants is central to engaging participants in study procedures and ensuring thorough and thoughtful data collection. Survey fatigue and general fatigue related to research participation pose real challenges to data collection as well as study retention [9]. Interactions with participants vary in length and frequency depending on study protocols; however, each interaction should be viewed as an opportunity to build rapport with participants. Strategies to build rapport include smiling (if on a video call), communicating clearly and confidently, and providing adequate emotional and technical support [5,11] (Figure 1). Researchers, clinicians, and patients alike cite increased mental health symptoms, stress, and added duties owing to the pandemic [22]. It is important to keep these additional burdens in mind when communicating with participants. Adjusting calls about study measures to be more conversational (eg, making time to ask participants about their day and how they are doing) can aid in establishing and maintaining rapport in the study team–participant relationship. The shared experience of COVID-19 is unifying and can be a source of common ground to relate to participants. Engaging in this way and expressing gratitude for participants’ time can help build participant investment in the study.

Study teams face additional challenges in prompting participants to complete measures when participants are difficult to reach or are unresponsive. Persisting in using creative outreach methods for calling and texting participants using HIPAA-compliant technologies (eg, Cisco Jabber and Twilio) [1] is essential. Study teams should consider adopting standardized procedures for attempted contact with participants to limit the burden on both participants and the study team. Often, research coordinators or research assistants are the first line of communication with participants and will attempt to call participants a certain number of times. It is helpful to consider when participants are usually home (ie, what time of the day is best to call) and to try different times throughout the day to achieve higher response rates. Study teams should standardize the maximum number of outreach attempts by research coordinators. Once that number is reached, it has proven useful in our experience to pass the communication up the chain to a study clinician or principal investigator. Study teams can also use this approach to allow a clinician to assess whether disengagement may be related to any concerns regarding the participant’s well-being. Other strategies to bolster participant motivation include involving family members in study procedures, accommodating participants’ preferred methods of communication (eg, texting, email, and phone call), and providing monetary or other forms of incentives [9,11].

### Promoting Health Equity and Overcoming Barriers to Web-Based Engagement Among Participants With Varying Levels of Technology Access and Familiarity

As the COVID-19 pandemic continues to lay bare the existing health disparities in racially, ethnically, and socioeconomically minoritized groups, concerns that the increased reliance on digital technologies for clinical care and research will exacerbate the digital divide rather than mitigate systemic health inequities are prevalent [23]. Indeed, digital access is considered a social determinant of health, with 21 million adults in the United States lacking access to broadband internet [24]. With the transition to web-based research, we risk compounding this structural disadvantage and not realizing the potential to expand research access to increasingly diverse and underrepresented populations [1] without targeted measures to address digital access and literacy [21,25,26].

Building capacity for person-centered, equitable research can be facilitated by providing smartphones or internet plans to participants if access to these technologies is an inclusion requirement [1,11] as well as using multiple outreach modalities. Enabling outreach through multiple modalities has led to successful data collection during the pandemic in our ongoing randomized controlled trial for patients with serious mental illness and a new cancer diagnosis [27]. In this trial, we use multiple traditional outreach methods for data collection (ie, phone, email, and letter mail) in addition to nontraditional methods such as partnering with family caregivers and staff in congregate living settings. Despite a slower study accrual because of fewer new oncology consultations during the pandemic, we maintained consent and survey completion rates for a marginalized population with flexible, multimodal, patient-centered outreach [28].

Providing adequate technology support is also of utmost importance. Study teams must provide training to participants for all forms of technology used, through manual documentation, prerecorded videos, or live assistance (eg, over the phone) [11]. Proactive outreach to individuals for technology coaching can promote efficiency and decrease participant frustration. Test-driving technologies and creating a short list of common technology challenges encountered by participants can help study teams troubleshoot and identify unnecessarily confusing aspects of instructions or procedures that can be changed. Study teams can also consider engaging family members in study procedures, which has been shown to aid in the adoption of technology for older populations with cognitive impairment [11]. We commonly use the approach of *meeting participants where they are* by first assessing participants’ comfort with technology during a study enrollment phone call. This allows us to provide extra support where necessary, such as detailed instructions on software installation, test calls with study staff, and encouragement. We also prioritize conducting qualitative exit interviews to obtain feedback on study procedures to refine study protocols and participant instruction materials [14]. Technical support activities may increase the total time spent both preparing for and conducting a session with a participant. However, the time invested in participants proactively will contribute to improved data quality by ensuring patient understanding of the technology and study measures. Furthermore, digital solutions tailored for specific populations can aid in realizing the potential for web-based research to increase accessibility to underrepresented individuals.

### Practical and Logistical Considerations to Conducting Qualitative Interviews and Focus Groups Remotely

Focus groups, or interviews, are conducted synchronously; therefore, time (and time zone) coordination is required. For individual interviews, offering flexible hours that prioritize participants’ preferences may assist in study enrollment because participants will be able to schedule and mark their calendars for a *study visit* in real time. To coordinate a focus group, study staff can ask participants about their availability within multiple potential time blocks to choose a time to maximize attendance. Once a specified time frame has the minimum target focus group size, study staff may call unavailable *participants* to assess whether there has been a change in schedule or continue recruiting to reach the maximum focus group limit, ranging anywhere from 4 to 12 participants [29], with smaller groups often preferred for web-based conduct. In general, participants should be made aware before the interview or group what the policies will be (ie, how long the group will run, expectations for video on or off, and audio-recording plan).

HIPAA-compliant videoconferencing software (eg, Zoom and WebEx) is necessary for the conduct of remote qualitative interviews or focus groups (as opposed to phone calls) to facilitate rapport building between study staff and participants to ensure that participants feel at ease. Many types of videoconferencing software contain features, such as waiting rooms and passcodes, that maximize participants’ security and confidentiality. Still, participants should be informed of the privacy risks associated with participation in remote focus groups (eg, the unsanctioned audiotaping or videotaping of groups) and the rules for participation (eg, use of headphones and being against recording of groups) should be clearly articulated at the start of every group. Features such as *breakout rooms* can also be innovatively used to conduct multiple interviews at one time, such as in the case of exit interviews after focus groups. Microphone and video camera positioning should be considered for both the interviewer and the interviewee, and 5 to 10 minutes should be allotted to ensure the proper placement and functioning of microphones and video cameras to enhance the quality of data. Automated live captioning of the interview conversation (closed captions) may also benefit participants who have difficulty hearing.

Having study staff on call during interviews and focus groups is essential to provide technological support to participants in case of issues. Study staff can provide individual support to participants and troubleshoot issues related to remote participation, including poor connectivity with the internet, audio or camera issues, the use of videoconferencing software, and environmental disruptions [11]. In the case of challenges that cannot be solved within a reasonable amount of time, study staff should have backup strategies in place to conduct interviews over the phone, allow participants to join focus groups by phone, or reschedule meetings flexibly. These procedures were used in qualitative interviews with patients with young-onset dementia and their caregivers [30], as well as in focus groups with orthopedic medical providers to enable the recruitment of geographically diverse participants.

We used these strategies at the beginning of the pandemic to transition from an in-person focus group study investigating barriers to smoking cessation clinical trials for Hispanic, Latino, or Latina individuals to remote procedures. Before the pandemic, we recruited Hispanic, Latino, or Latina individuals for focus groups conducted in both English and Spanish. After transitioning to remote research, we ran the web-based focus groups with smaller numbers than intended in person (3-4 people) to ease the burden on the study team while we navigated the new technology and ensured that each participant was able to receive one-on-one assistance. We faced challenges with technology, including finding solutions for individuals who did not have email or webcam access, a noted disparity among older Hispanic individuals [31]. To increase access, we mailed information to all participants (eg, study information sheet and materials to be discussed during the group) 1 week before the group and expanded our protocol to include both telephone conference calls and videoconferencing calls to accommodate participants’ varying levels of technology access. Despite technological challenges, we found that offering web-based focus groups was helpful for both participants and study staff because we could more flexibly schedule groups with the bilingual study staff member who facilitated the groups. We also offered participants the option to have a *test call* with a member of the study staff to ensure adequate internet connection, microphone or camera functioning, and confidence navigating the video software. An alternative method would be to include a brief introduction to the video software at the beginning of a qualitative interview or focus group and encourage participants to test different functions (eg, toggling audio and video on and off).

### Adapting Facilitation Strategies for Remote Qualitative Data Collection

Although remotely conducted interviews and focus groups may pose some challenges to interviewers in engaging participants, connecting with participants, and encouraging open and active dialogue among participants, there are many verbal and nonverbal strategies that interviewers can adopt. Before the interview, study staff should begin building rapport with participants (Figure 1), explain who will be conducting the interview with their credentials, and provide information on what topics the interview will cover (particularly important for sensitive topics). At the start of the interview or focus group, interviewers should warmly introduce themselves and provide additional reminders to set the appropriate tone. For example, interviewers should encourage participants to be in a quiet and private space (or use headphones) with efforts to minimize environmental distractions (eg, participants should not be driving, doing chores, or eating) [11]. Interviewers may want to encourage participants to keep their camera on if they are able to facilitate engagement and rapport building but to mute themselves when they are not talking to reduce background noise. If participants are muted, interviewers should be prepared to probe them more enthusiastically than usual to motivate active dialogue and participation. It may be helpful for interviewers to continually encourage participants to share, particularly those who have been quiet. Encouraging diversity of opinion among groups can also help participants feel comfortable expressing their personal experiences and differing perspectives.

Assuming that they are visible to participants, interviewers should also pay attention to their nonverbal communication. If interviewers must take notes during qualitative data collection and are therefore unable to maintain eye contact throughout the interview or focus group, participants should be informed to avoid potential nonverbal miscommunication. Reactive facial expressions are critical in remote qualitative data collection, as body language cannot be observed as it typically would be in person, although some aspects such as posture may be observed. Nonverbally reacting appropriately to what participants share is vital to encourage participants to be open and honest during an interview. The key aspects of nonverbal communication include eye contact (toward the participant or the camera), using facial expressions to demonstrate understanding and listening, and body language, including nodding [11].

For structured and semistructured interviews and focus groups, keeping track of the timing during the interview is also necessary to ensure that all questions are answered, with appropriate time allocated to each section or question. This is particularly important for remotely conducted interviews, in which participants may only reserve the exact expected amount of time for the call (eg, 60 minutes) and when adequate attention and focus might be more difficult to maintain than in person. To support interviewers in managing time, we commonly include time stamps in interview guides and denote the questions to be prioritized. In focus groups, it is recommended to have 2 interviewers on the call if possible. That way, at least one interviewer can be primarily concerned with active listening and engagement with the participants, whereas another interviewer can focus on note-taking and timekeeping.

In our recent qualitative study with patients with young-onset dementia and their caregivers (dyadic interviews), we found it critical to consider the specific cognitive challenges of persons with dementia in facilitation as well as the sensitive nature of dyadic interviews. All questions were piloted with experts in young-onset dementia before the interviews to ensure clarity. Interviewers were prepared to repeat questions several times as well as define or explain keywords as needed. Because couples were asked to share their perspectives regarding the person with dementia’s symptoms and illness progression as well as relationship satisfaction in front of each other, we prefaced the interview by validating the difficulty of openly sharing and encouraging participants to be as open as possible. When participants were hesitant in sharing, we found that *sitting with the silence* before moving on to a new question encouraged participants to reflect and add to the conversation. Before asking about relationship challenges, the interviewer acknowledged that this might be the first time couples are discussing certain questions and assured couples that we would be available to provide support to the couple together or individually after the interview as well. It is particularly important to consider participant emotional safety and sense of support in the case of remote interviews.

### Essential Training Competencies for Study Staff

At the forefront of training competencies to conduct remote data collection is ensuring study staff have familiarity with practices to promote participant privacy and security, including encrypting computer devices; using secure, encrypted video and audio software; and conducting qualitative data collection in private, quiet locations. Equipping the study team with institutionally encrypted equipment (laptops with webcams and phones) and software programs facilitates standardized and HIPAA-protected data collection [1]. It is essential that study staff have sufficient familiarity with all technologies used so that they can troubleshoot any problems that may arise for either themselves or the participants and provide technical support as needed [11]. Therefore, study staff must be thoroughly trained in the use of any relevant technology as well as provided with resources to contact in the case of questions or issues.

Given the unique challenges to rapport building and participant engagement through remote encounters, it is also important to provide study staff with adequate training in verbal and nonverbal communication. For study staff with less experience with participant interaction and without clinical training, providing some level of peer or hierarchical supervision may be helpful in supporting them in developing effective communication skills.

## Discussion

### Summary

In this paper, we integrated recommendations from previous literature with examples from our ongoing clinical research to identify and respond to specific challenges to remote data collection (Tables 1 and 2). We hope to catalyze other research teams to think critically about the strategies they use in remote data collection and contribute to the collective body of knowledge on best practices through the publication of protocol papers and other methodologically oriented works. It is imperative that research teams thoughtfully and creatively solve problems in response to the challenges they face in remote data collection to ensure the validity and quality of data as well as the patient-centeredness of study procedures.

### Future Directions

Future research is needed to evaluate whether data collected through web-based study designs are of the same nature and quality as data collected through traditional in-person approaches and to continue to identify strategies to maximize the validity of data collected remotely. The shift toward more web-based designs prompted by the COVID-19 pandemic brings with it the opportunity to remove many barriers of access to clinical research and engage more diverse participant populations while minimizing the burden on participants. However, without proper capacity building for web-based research, we risk widening the digital divide perpetuating existing disparities. We discussed our experiences with conducting web-based research with different populations, including individuals underrepresented in research such as Hispanic, Latino, or Latina individuals, those with serious mental illness, and those who face increased barriers to research participation, such as older adults with dementia and adolescents with learning disabilities. The strategies presented (eg, device provision, increasing technological support, and using multiple modalities to conduct research) are examples of mechanisms to promote equity in research participation. We acknowledge the significant participant burden in using technology for research and that the same digital health solutions do not work for all individuals. Therefore, it is imperative that researchers assess barriers specific to their study designs and populations of interest to mitigate the threat of increasing existing disparities. Additional research is needed to further characterize strategies that can be used to ensure accessibility of virtually conducted research to marginalized and underrepresented populations.

## Abbreviations

6MWT: 6-minute walk test
HIPAA: Health Insurance Portability and Accountability Act
REDCap: Research Electronic Data Capture
